# Correlated Target Search by Vaccinia Virus Uracil–DNA Glycosylase, a DNA Repair Enzyme and a Processivity Factor of Viral Replication Machinery

**DOI:** 10.3390/ijms24119113

**Published:** 2023-05-23

**Authors:** Evgeniia A. Diatlova, Grigory V. Mechetin, Anna V. Yudkina, Vasily D. Zharkov, Natalia A. Torgasheva, Anton V. Endutkin, Olga V. Shulenina, Andrey L. Konevega, Irina P. Gileva, Sergei N. Shchelkunov, Dmitry O. Zharkov

**Affiliations:** 1 SB RAS Institute of Chemical Biology and Fundamental Medicine, 8 Lavrentieva Ave., 630090 Novosibirsk, Russia; e.diatlova@g.nsu.ru (E.A.D.); mechetin@niboch.nsc.ru (G.V.M.); ayudkina@niboch.nsc.ru (A.V.Y.); ashatan.314@gmail.com (N.A.T.); aend@niboch.nsc.ru (A.V.E.); 2 Biology Department, Tomsk State University, 634050 Tomsk, Russia; arthropodae01@gmail.com; 3 NRC “Kurchatov Institute”—B. P. Konstantinov Petersburg Nuclear Physics Institute, Leningrad Region, 188300 Gatchina, Russia; ovshulenina@gmail.com (O.V.S.); konevega_al@pnpi.nrcki.ru (A.L.K.); 4 State Research Center of Virology and Biotechnology Vector, Novosibirsk Region, 630559 Koltsovo, Russia; gileva@vector.nsc.ru (I.P.G.); snshchel@rambler.ru (S.N.S.); 5 Department of Natural Sciences, Novosibirsk State University, 2 Pirogova St., 630090 Novosibirsk, Russia

**Keywords:** DNA repair, uracil–DNA glycosylase, protein translocation, viral replication, processivity, correlated cleavage, random walk, vaccinia virus, protein targeting

## Abstract

The protein encoded by the vaccinia virus *D4R* gene has base excision repair uracil–DNA *N*-glycosylase (vvUNG) activity and also acts as a processivity factor in the viral replication complex. The use of a protein unlike PolN/PCNA sliding clamps is a unique feature of orthopoxviral replication, providing an attractive target for drug design. However, the intrinsic processivity of vvUNG has never been estimated, leaving open the question whether it is sufficient to impart processivity to the viral polymerase. Here, we use the correlated cleavage assay to characterize the translocation of vvUNG along DNA between two uracil residues. The salt dependence of the correlated cleavage, together with the similar affinity of vvUNG for damaged and undamaged DNA, support the one-dimensional diffusion mechanism of lesion search. Unlike short gaps, covalent adducts partly block vvUNG translocation. Kinetic experiments show that once a lesion is found it is excised with a probability ~0.76. Varying the distance between two uracils, we use a random walk model to estimate the mean number of steps per association with DNA at ~4200, which is consistent with vvUNG playing a role as a processivity factor. Finally, we show that inhibitors carrying a tetrahydro-2,4,6-trioxopyrimidinylidene moiety can suppress the processivity of vvUNG.

## 1. Introduction

Base excision DNA repair (BER) is an important protective pathway keeping the genomes of living cells free of deaminated, oxidized, and alkylated bases, apurinic/apyrimidinic (AP) sites, and single-strand breaks [1,2]. When a damaged base is present, BER is initiated by one of several DNA glycosylases, the enzymes that specifically recognize a damaged base and hydrolyze its *N*-glycosidic bond [2,3]. The formed AP site is then processed via several subpathways involving AP endonucleases, DNA polymerases, DNA ligases, and a host of accessory proteins. DNA glycosylases usually possess general specificity for purine or pyrimidine bases damaged in chemically similar ways, e.g., for oxidized purines or ring-alkylated purines [1,2,3]. In their search for sites of DNA damage, DNA glycosylases employ the facilitated diffusion mechanism (often termed “processive search”) based on association with non-specific DNA and random one-dimensional diffusion to survey a short segment (tens to hundreds of nucleotides) for the presence of a lesion [4,5,6]. The opposite of processive search is distributive search, where the protein binds and releases DNA without significant lateral movement until it happens to encounter the target.

Uracil, one of the most abundant types of DNA damage, arises from cytosine deamination, either spontaneous or catalyzed by cytosine deaminases, and appears in the genome through either direct damage to C in DNA or incorporation of dUMP from the pool of residual metabolic dUTP or damaged dCTP [7,8]. Hydrolytic C deamination is unavoidable in aqueous solution and thus affects every living being. U is removed from DNA through the action of uracil–DNA glycosylases (UNG), the enzymes universally found throughout cellular life and in some viruses with DNA genomes. Human cells, for example, possess four enzymes capable of U excision: UNG, single-strand selective monofunctional uracil–DNA glycosylase (SMUG1), G/T mismatch-specific thymine–DNA glycosylase (TDG), and methyl-CpG-binding domain protein 4 (MBD4). Of these, UNG, SMUG1, and TDG belong to the same structural superfamily sharing the α/β-fold. UNG, which seems to be the primary U-removing enzyme, is associated with the replication fork and is cell-cycle-regulated, while SMUG1 likely contributes to the protection of the genome from U formation outside of the S phase [8,9].

While UNG superfamily members can be found in nearly every cellular genome sequenced so far, the role of these enzymes and U repair in general in the life cycle of viruses remains poorly characterized. UNG homologs are widely present in the genomes of herpesviruses, poxviruses, many giant viruses (e.g., Mimivirus, Pandoravirus, and Pithovirus), and a limited number of bacteriophages. However, the necessity of UNG for replication or infectivity was only demonstrated for some herpesviruses [10,11,12,13] and poxviruses (see below). Viruses, of course, can subvert host cell DNA repair systems for their own use; this strategy is used by human immunodeficiency virus 1 which incorporates host UNG into the viral particles to protect its cDNA genome from mutations [14,15,16].

Poxviruses are among a few groups of viruses requiring the presence of uracil–DNA glycosylases for their replication. Poxviruses encode their own UNG protein (*D4R* gene in the vaccinia virus strain Copenhagen genome; below, we refer to the vaccinia virus protein as vvUNG), and its deletion drastically decreases the ability of the virus to replicate in cells [17,18,19]. The enzymatic properties of vvUNG and its close relative, monkeypox virus UNG, have been partially characterized, revealing fairly typical UNG kinetics, substrate specificity, and buffer requirements but resistance to the inhibiting action of Ugi, a small phage protein tightly binding to UNG [20,21]. The structure of vvUNG has been solved both alone and bound to normal DNA, damaged DNA, and viral A20 protein that does not bind DNA but tethers vvUNG to the viral replication complex [22,23,24,25,26,27,28,29,30].

There are conflicting data as to whether the viability of poxviruses depends on the repair function of their UNG. Some studies reported that selective inactivation of DNA glycosylase function by site-directed mutagenesis leads to suppression of replication [19], while others claim that the replication is supported by mutant forms of the enzyme lacking catalytic activity [31]. It is known that UNG-deficient vaccinia virus can only replicate in cells ectopically expressing vvUNG but not the host UNG [32,33]. The virulence of vaccinia virus strain carrying mutations in the vvUNG active site in mice is sharply reduced [31]. It has been suggested that, instead of DNA repair, the main role of UNG in poxviral replication may be imparting processivity to viral DNA polymerase, since the heterodimer of DNA-binding D4 and bridging A20 proteins acts a processivity subunit of the poxvirus DNA replication complex [23,34]. However, the processivity of vvUNG or UNG from any other poxviral species has never been studied. Notably, structures of vvUNG indicate that, unlike PolN/PCNA sliding clamps, it does not fully close around DNA, so it is unclear whether its intrinsic processivity can support viral replication.

To assess the processivity of BER enzymes in a quantitative way, we [35] and others [36,37] independently developed a method for kinetic analysis based on the probability of correlated cleavage (*P*_cc_; see Supplementary Text for a formal description) of substrates containing a pair of damaged sites separated by variable distances. Essentially, the probability of transfer between two sites without releasing DNA is taken as a measure of processive lesion search. Using a random walk model on a one-dimensional lattice (e.g., [38]), one can then estimate the biologically relevant parameters such as the average survey length. Since then, the approach has been applied to estimate the processivity of human and *E. coli* UNG (hUNG and EcoUng, respectively), 8-oxoguanine–DNA glycosylases Fpg and OGG1, alkylpurine–DNA glycosylases AlkA and MPG, and AP endonucleases Nfo and APEX1 [35,36,37,39,40,41,42,43,44,45,46,47,48,49,50,51,52,53]. In this work, we quantitatively characterize the lesion search by vvUNG and conclude that its intrinsic ability for one-dimensional diffusion along DNA is indeed compatible with the role of this protein as a polymerase processivity factor.

## 2. Results

### 2.1. vvUNG Is Capable of Correlated DNA Cleavage

Correlated cleavage of substrates containing two U bases reflects the ability of UNG enzymes to move from one lesion to another without releasing the bound DNA [35,37]. In DNA-binding proteins, the physical basis of such movement can be either movement along the helical axis without losing the essential contacts with DNA (sliding) or short-distance dissociation/association events without bulk rehydration of the protein–DNA interface (hopping) [54,55,56]. In any case, the correlated search characteristically depends on the ionic strength of the solution, reflecting the mostly electrostatic nature of protein–DNA interactions and the need to expel bound ions from the interface upon lateral movement.

**Figure 1:**
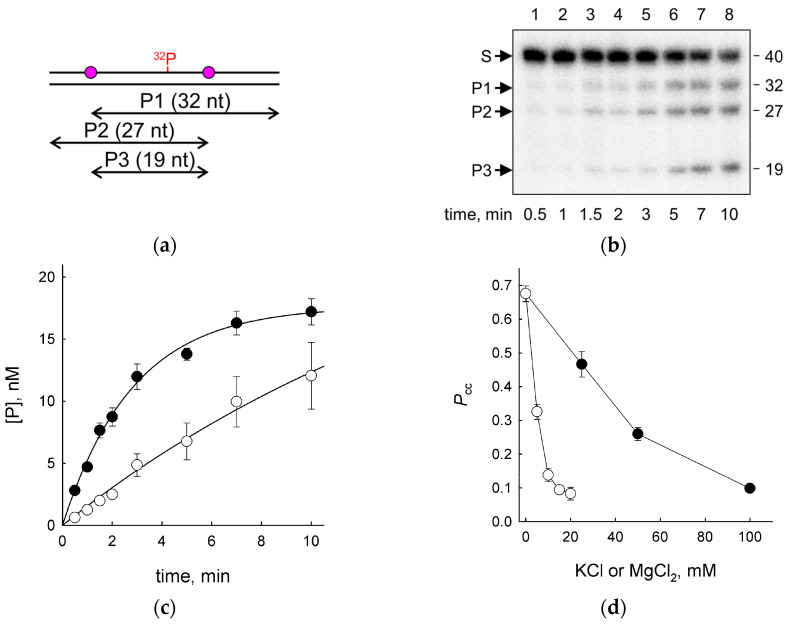
Correlated cleavage of DNA carrying two U bases by vvUNG. (**a**) Scheme of the substrate. Purple circles correspond to the lesions; ^32^P marks the position of the radioactive label. P1, P2, and P3 are the cleavage products with their respective lengths. (**b**) Representative gel image showing the accumulation of cleavage products with time. Arrows: S, substrate; P1–P3, cleavage products as in Panel A. Lengths of the substrate and the products are marked next to the gel. (**c**) Time course of product accumulation (25 mM KCl). Closed circles, P1 + P2; open circles, P3. (**d**) Dependence of *P*_cc_ on the concentration of KCl (closed circles) and MgCl_2_ (open circles). Mean ± SD of three independent experiments is shown in (**c**,**d**).

Using a double-stranded substrate with two U residues located in the identical sequence context and separated by 19 nt of undamaged intervening sequence, we followed the accumulation of products cleaved at one site and at both sites (Figure 1a–c). For the correlated cleavage experiments, a large excess (>400-fold) of the substrate was taken to minimize independent cleavage at both sites, and only the linear parts of the product accumulation time courses were considered to ensure steady-state conditions. The efficiencies of U excision from 20-mer duplex substrates corresponding to each individual half of the construct were determined in separate experiments and were found to be similar (Supplementary Figure S1; U20L//G20L: *K*_M_ = 400 ± 140 nM, *k*_cat_ = 5.8 ± 0.8 s^−1^; U20R//G20R: *K*_M_ = 330 ± 80 nM, *k*_cat_ = 7.3 ± 0.5 s^−1^). When the reaction mixture contained only 25 mM Tris–HCl (approx. 20 mM in monovalent cations at 37 °C and pH 7.5) and low concentrations of EDTA and DTT, about 70% of cleavage events at one site were followed by cleavage at the second site (Figure 1d). However, a gradual increase in salt (KCl) concentration up to 200 mM drastically decreased *P*_cc_, consistent with the mechanism dependent on protein one-dimensional diffusion along DNA (Figure 1d). Notably, vvUNG showed higher *P*_cc_ values than EcoUng at any KCl concentration (Supplementary Figure S2). Substituting MgCl_2_ for KCl had an even larger effect, with the cleavage being almost fully distributive already at 10 mM (Figure 1d). This is not surprising given the much tighter binding of Mg^2+^ ions to DNA compared with monovalent cations. Even when the total ionic strength of the solution was taken into account, MgCl_2_ still was more detrimental for the correlated cleavage (Supplementary Figure S3a). This effect was not due to better stability of the duplex in the presence of divalent cations, since when *P*_cc_ was plotted against the equivalent cation concentration (i.e., the concentrations of Mg^2+^ and K^+^ that stabilize duplex DNA equally [57]), MgCl_2_ had less influence on the correlated cleavage than KCl (Supplementary Figure S3b). Thus, the more pronounced effect of MgCl_2_ is likely due to its tighter binding to DNA that complicates Mg^2+^ displacement by the moving protein.

**Figure 2:**
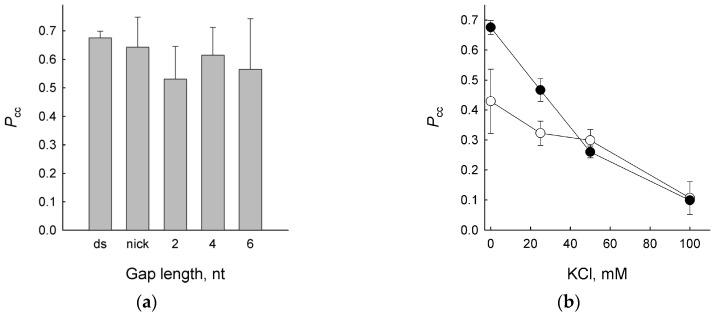
Correlated cleavage by vvUNG with nicks or gaps (**a**) or a bulky fluorescein residue (**b**) between two U residues. Mean ± SD of three independent experiments is shown. In (**a**), ds, uninterrupted duplex; nick, duplex lacking a single phosphate. In (**b**), closed circles are *P*_cc_ values for the internally ^32^P-labeled substrate, open circles are *P*_cc_ values for the fluorescein-labeled substrate.

UNG enzymes, including vvUNG, are known to excise U from single- and double-stranded DNA [20,58,59]. Moreover, the ability of EcoUng to perform correlated cleavage of a double-stranded substrate is not compromised by intervening single-stranded gaps up to 6 bp in length [41]. We inquired into whether vvUNG can bypass nicks and short gaps (2, 4, or 6 nt) without releasing DNA. The values of *P*_cc_ were similar in all cases (Figure 2a), indicating that single-stranded DNA gaps do not represent an obstacle for moving vvUNG.

Introduction of a bulky group such as fluorescein into the DNA duplex interferes with the one-dimensional diffusion ability of EcoUng [52]. Replacing the [^32^P]-phosphate label between the damaged sites with a fluorescein moiety tethered to T through an aminohexyl-3-acrylimide linker significantly reduced the *P*_cc_ of vvUNG at low salt concentrations but had a much less pronounced effect at ≥50 mM KCl (Figure 2b). This observation is consistent with the dominating effect of a sliding mechanism of vvUNG at low salt.

We also compared the correlated cleavage in substrates containing 19 or 20 undamaged nucleotides between the lesions as a check for the effect of the rotation phase around the helical axis. If the moving protein tracks a DNA groove, the differences in *P*_cc_ will be minimal, whereas if it moves along the linear axis, one could expect larger differences due to the helical twist that displaces the adjacent nucleotides by 36° in B-DNA [60]. The difference between the *P*_cc_ values was indeed minor (0.67 ± 0.02 for the 19 nt distance, 0.65 ± 0.05 for the 20 nt distance), again consistent with the movement along the DNA groove.

### 2.2. vvUNG Binds Damaged and Undamaged DNA with Similar Affinity

**Figure 3:**
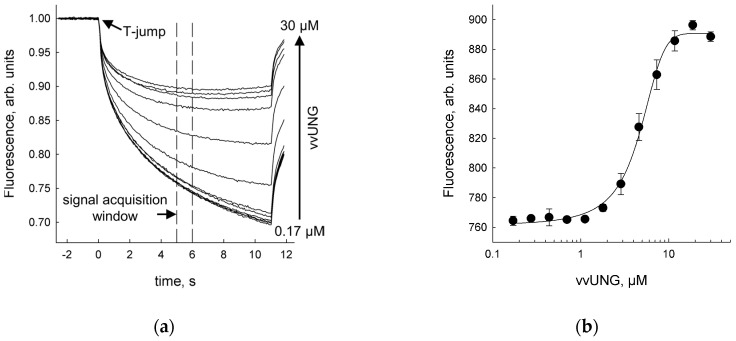
Binding of vvUNG to DNA followed by microscale thermophoresis. (**a**) Representative set of fluorescence traces for the C:G ligand. (**b**) Binding curve for the C:G ligand. Mean ± SD of three independent experiments is shown.

To clarify the origins of efficient transfer of vvUNG along DNA, we have used microscale thermophoresis to measure the affinity of vvUNG for single- and double-stranded DNA, both undamaged and containing (3-hydroxytetrahydrofuran-2-yl)methyl phosphate (F), an AP site analog mimicking the product of the DNA glycosylase reaction. Binding of vvUNG to oligonucleotide ligands 5′-labeled with the Cy3 dye produced clearly visible changes in the thermal diffusion of the complex (Figure 3a). The affinity of vvUNG for single-stranded undamaged DNA, double-stranded undamaged DNA, F-containing single-stranded DNA, and double-stranded DNA with F:G or F:A pairs was in the same order of magnitude, ranging from 4 μM to 12 μM (Figure 3b, Table 1). F-containing ligands were bound somewhat better, but the preference for the damaged DNA was at most threefold (Table 1) indicating that vvUNG possesses a rather uniform ability to bind any DNA, as would be expected of a processivity factor.

The *K*_d_ values found here are severalfold higher than those reported for EcoUng non-specific binding to double-stranded DNA (1–1.5 µM) [37,61]. However, since our experiments were performed at higher salt concentrations, with a shorter duplex, and using a different assay, it can be assumed that the general binding affinities of vvUNG and EcoUng for non-specific DNA duplex are comparable.

### 2.3. Efficiency of Lesion Recognition by vvUNG

**Figure 4:**
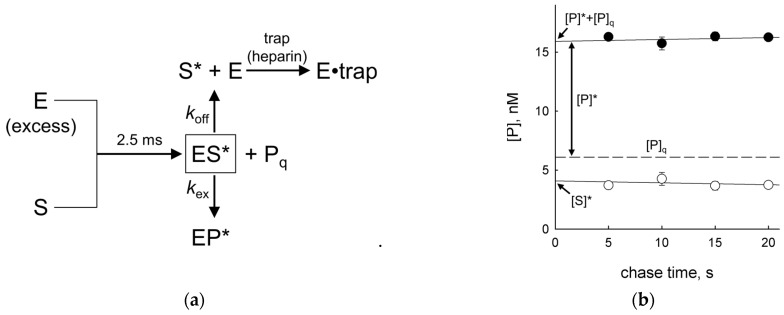
Excision efficiency of vvUNG measured in quench flow experiments. (**a**) General scheme of the experiment. (**b**) Accumulation of the product (closed circles) and consumption of the substrate (open circles) during the chase. P_q_, product formed within 2.5 ms; P*, product formed during the chase period; S*, substrate remaining during the chase period. Mean ± SD of three independent experiments is shown.

The probability of correlated cleavage *P*_cc_ is a product of two factors: the probability of transfer from one site to another (*P*_T_) and the probability of U excision upon encounter (*P*_E_) [37]. Thus, to single out the transfer probability, we determined *P*_E_ in a separate pulse–chase kinetic partitioning experiment. The scheme was essentially as described in [37,43] for hUNG and EcoUng with minor modifications: we mixed vvUNG (2 μM) with the substrate (20 nM) in a rapid quench flow apparatus and 2.5 ms later either quenched the reaction with alkali to obtain the amount of the product already formed at the first technically achievable quench time, or chased with a trap (heparin) for up to 20 s (Figure 4a). After the trap was added at 2.5 ms, all the product formed later was derived in a single-turnover mode from the ES complex existing by that time. Extrapolation of the product accumulation to zero time, corrected for the amount of product already formed at 2.5 ms (6.1 ± 1.0 nM in our case), gave the amount of product formed from a single lesion-binding event. The ratio of this product ([P]*; 10.6 ± 1.1 nM) to the extrapolated zero time substrate ([S]*; 3.3 ± 0.3 nM) gave the ratio of the glycosidic bond cleavage rate (*k*_ex_) to the non-productive dissociation rate of the ES complex (*k*_off_) [37] (Figure 4b). From this, the excision probability *P*_E_ may be obtained as *P*_E_ = *k*_ex_/(*k*_ex_ + *k*_off_) = [P]*/([P]* + [S]*). For vvUNG, it was 0.76 ± 0.02, which is very close to the *P*_E_ = 0.73 and 0.81 reported for EcoUng and hUNG, respectively [37,43]. This value was taken to calculate *P*_T_ from *P*_cc_ in the modeling exercise described in the next section.

### 2.4. Dependence of P_cc_ on the Distance between the Lesions

**Figure 5:**
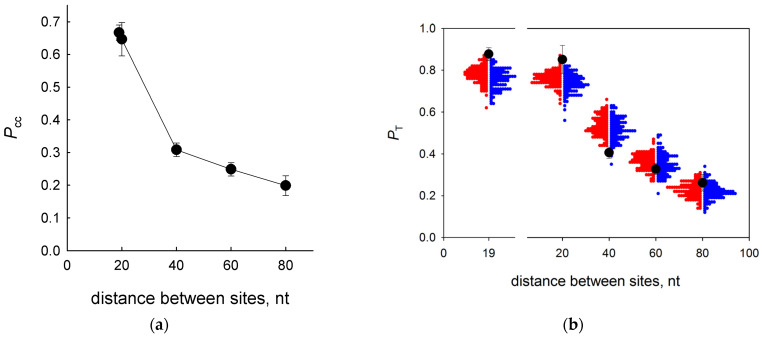
(**a**) Dependence of vvUNG *P*_cc_ on the distance between the U residues. Mean ± SD of three independent experiments is shown. (**b**) Simulation of vvUNG random walk on a finite one-dimensional grid with irreversible losses. Black symbols are experimental *P*_T_ data used for fitting. Colored dots show the fraction of successful walks from position 8 to positions 28, 29, 49, 69 or 89 (red dots) or from positions 28, 29, 49, 69 or 89 to position 8 (blue dots).

In order to estimate the effective survey distance using vvUNG, we have constructed a series of substrates in which two U residues were separated by non-damaged intervening sequences of different lengths (19, 20, 40, 60, or 80 normal nucleotides between the lesions). When conducting the search, the enzyme could fall off the DNA either from an internal position or from one of the ends, and the fraction of the enzyme that reached the second site decreased with wider separation between the lesions [38,43]. Indeed, the efficiency of the intersite transfer dropped sharply when the distance increased from 20 nt to 40 nt and then continued to decrease less sharply, in total descending from 0.67 ± 0.02 at a distance of 19 nt to 0.20 ± 0.03 at a distance of 80 nt (Figure 5a). Notably, these *P*_cc_ values were always higher than the values for EcoUng obtained in the same substrate system under the same conditions [41] where *P*_cc_ dropped from 0.39 with 20 nt separating the damaged sites to 0.12 with 80 nt separating the damaged sites (Supplementary Figure S4).

In previous studies employing the two-site cleavage assay, estimates of the biologically relevant parameters of EcoUng one-dimensional diffusion were made analytically using available models of a one-dimensional random walk [37,41,43]. Here, we used an alternative approach, namely the simulation of a one-dimensional random walk with losses, to evaluate the microscopic characteristics of vvUNG processivity. We employed a simple model of a single-node-size particle walking in discrete steps along a finite one-dimensional grid, with each step carrying a probability of irreversible loss from an internal node *p*_off_ and a larger probability of irreversible loss from a terminal node *p*_off_ + *p*_end_. The estimates of *p*_off_ and *p*_end_ were made from fitting simulated walks on grids whose lengths and start and finish node position corresponded to the actual substrates (~3 × 10^9^ walks in total) to the experimental *P*_T_ data (see Materials and Methods for a detailed description). Initially, we benchmarked the simulations against the experimental data for EcoUng and the estimates made from these data using the Belotserkovskii–Zarling analytical approximation of a random walk with losses [38]. The simulation produced the values of *p*_off_ = (4.99 ± 0.64) × 10^−5^ and *p*_end_ = 0.179 ± 0.004 (mean ± s.e.m.), in reasonable agreement with *p*_off_ ~ 1 × 10^−4^ and *p*_end_ = 0.17 as reported in [41] (Supplementary Figure S5). The distribution of successive walks in the opposite directions was notably asymmetric due to high *p*_end_ and different lengths between the ends and the start and finish nodes (Supplementary Figure S5). Global fitting of the simulation to the experimental data for vvUNG (Figure 5b) quite unexpectedly produced a ~5-fold higher *p*_off_ = (2.41 ± 0.07) × 10^−4^ and a ~30-fold lower *p*_end_ = (6.06 ± 0.33) × 10^−3^ (mean ± s.e.m.) in comparison with EcoUng. This, in particular, may indicate that the physical processes underlying the off-rate estimates for EcoUng from *K*_d_ experiments with short non-specific oligonucleotide substrates [37,61] could be different from those for DNA release by vvUNG. Notably, the model underestimated *P*_cc_ at the shortest intersite distances, indicating the possibility of enhanced transfer at the ~20-nt range. However, alternative walk models that also entailed short-distance hopping or pauses in sliding produced quantitatively similar results, so the nature of this enhanced transfer remains to be established.

### 2.5. Small-Molecule Inhibitors Affecting Correlated Cleavage by vvUNG

**Figure 6:**
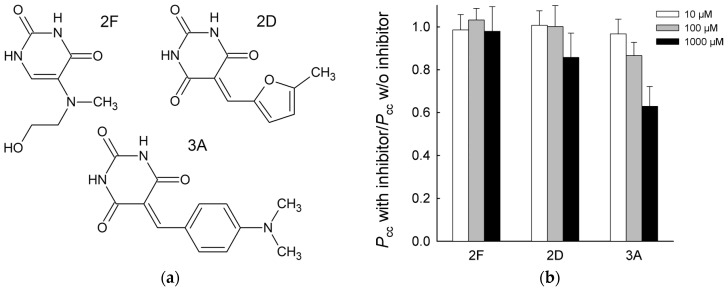
Inhibition of correlated cleavage by PyO3 derivatives. (**a**) Structures of the compounds 2D, 2F, and 3A. (**b**) Dependence of *P*_cc_ (normalized for *P*_cc_ without the inhibitor) on the inhibitor concentration. Mean ± SD of three independent experiments is shown.

Recently, we identified a series of low-molecular-weight compounds with modest inhibitory properties (IC_50_ ~ 10–100 μM) towards vvUNG [62]. These inhibitors are derived from the tetrahydro-2,4,6-trioxopyrimidinylidene (PyO3) moiety and, according to molecular docking, occupy the uracil-binding pocket of UNG and form additional contacts near its entrance. Here, we addressed the effect of these inhibitors on correlated cleavage by vvUNG. Since *P*_cc_ is calculated as a ratio of initial reaction rates, a decrease in *v*_0_ at the individual U sites due to inhibitor binding does not affect *P*_cc_, and any observable effect is due to a change in the intersite transfer efficiency. We used three compounds from the screened library (Figure 6a), of which compound 2F demonstrated no inhibitory properties, whereas compounds 2D and 3A were inhibitors (IC_50_ ~ 120 μM and 70 μM, respectively) [62]. No change in *P*_cc_ was observed with 2F up to 1000 μM. 2D caused a ~15% decrease in *P*_cc_ at the highest concentration used (1000 μM; Figure 6b). The effect of 3A was more pronounced: *P*_cc_ dropped by ~15% at 100 μM and by 37% at 1000 μM compound 3A (Figure 6b). Although the magnitude of the effect is moderate, these results show that intersite transfer can be suppressed by low-molecular-weight compounds that presumably compete with DNA for contacts in or near the enzyme’s lesion-binding site.

## 3. Discussion

DNA glycosylases are key members of the network safeguarding the genome from endogenous and environmental damage. There are rare occurrences of DNA glycosylases that, while maintaining the same catalytic chemistry, have evolved to play a role in other cellular processes. The best-known examples are thymine–DNA glycosylases (TDG) in vertebrates and DEMETER-like 5-methylcytosine glycosylases in higher plants, which excise epigenetically modified pyrimidine bases and mainly participate in gene activity regulation and chromatin organization [63,64]. There is increasing evidence that mammalian 8-oxoguanine–DNA glycosylase OGG1 can serve as a transcription activator and a guanine nucleotide exchange factor for some regulatory small GTPases [65]. However, the role of vvUNG in the viral replication complex so far appears to be unique among DNA glycosylases.

In the vast majority of living organisms, the replication machinery relies on dimeric or trimeric doughnut-shaped clamps to ensure processive DNA synthesis [66]. In herpesviruses, UL42 and UL44 processivity factors are, respectively, monomers and dimers and do not fully encircle DNA but share the same “processivity fold” with canonical clamp proteins [67,68]. Other than vvUNG, the only known DNA polymerase processivity factor not belonging to the clamp superfamily is thioredoxin, a bacterial protein adopted as a processivity subunit by bacteriophage T7 DNA polymerase. Thioredoxin, however, does not seem to contact DNA directly but rather stabilizes a long polymerase loop that tracks along DNA [69,70,71,72]. Additionally, single-strand binding proteins, although not considered processivity subunits in a strict sense, often enhance the processivity of DNA polymerases and have been used toward this end in fusion constructs [73,74]. Moreover, fusions with non-specific DNA-binding protein Sso7d [75] and DNA-binding helix–hairpin–helix motifs [76,77] can improve DNA polymerase processivity. Almost all studied DNA glycosylases have the ability to move along DNA randomly in search of the damaged sites, yet only vvUNG is known to be important for replicative processivity. Thus, it was interesting to address the intrinsic processivity of vvUNG and see whether it is compatible with its role in the viral replication complex.

Single-molecule assays such as the tightrope assay have recently gained popularity in studies of the facilitated diffusion of DNA-binding proteins. However, the actual observed species in single-molecule experiments is usually not a protein molecule per se but a complex coupled to a quantum dot through an antibody, or a fusion with a fluorescent protein tag, which may skew the parameters obtained with relatively small proteins such as DNA glycosylases. Moreover, the DNA used in some types of such experiments is heavily saturated with an intercalating or minor groove binding dye for visualization, which may affect protein–DNA interactions. Last but not least, precise chemical modification of the substrate is complicated with long DNA used in single-molecule assays. Ensemble kinetic methods, while providing less direct access to the walk parameters, may better reflect the intrinsic properties of the proteins under study and easily allow the introduction of nicks, gaps, obstacles, and other substrate modifications useful for mechanistic studies. The glycosylase activity of vvUNG allowed us to apply a two-site cleavage kinetic assay to characterize the protein’s diffusion along DNA.

Overall, we found that vvUNG behaves as expected for a processive DNA glycosylase and is more processive than EcoUng: its *P*_cc_ was higher at the same KCl concentrations, and at the same intersite distances (Supplementary Figures S2 and S4). Similarly to EcoUng, vvUNG was able to traverse nicks and short gaps, most likely because its affinity for ssDNA and dsDNA was similar. Modeling the one-dimensional walk with the experimentally determined kinetic parameters arrived at a ~2.4 × 10^−4^ per-step probability of enzyme loss, corresponding to a mean lifetime of −1/ln(1 − *p*_off_) ≈ 4200 steps per association. If the movement is unidirectional, as in a replication complex performing DNA synthesis, this will be equal to the mean displacement, or the average number of added nucleotides per association. Isolated vaccinia virus DNA polymerase is nearly distributive (≤10 nt incorporated per binding event in 1 mM MgCl_2_ or 40 mM NaCl) but, complete with the A20 and vvUNG subunits, it can replicate over a ~7000 nt template in a single binding event [34,78,79]. Thus, the intrinsic processivity of vvUNG is in the same order of magnitude as the processivity of the full viral replication complex. It is quite possible that the fully assembled complex is more processive than its individual subunits. Assuming that the full complex is lost when both polymerase and vvUNG subunits are detached from DNA simultaneously, and roughly estimating the per-step *p*_off_ for the isolated DNA polymerase at ~0.2 (as (1 − 0.2)^10^ ≈ 0.1, this would correspond to ~10% polymerase molecules remaining bound after 10 nt incorporation and to ~4–5 nt being incorporated per association), the combined *p*_off_ = *p*_off_(vvUNG) × *p*_off_(pol) = 4.94 × 10^−5^, which translates to an average of ~21,000 nucleotides per association. It should also be taken into account that, although the probability-based description of a random walk is by definition time-independent, *p*_off_ actually depends on the mean dwell time of a step (ultimately determined by the diffusion rate). In the replicative complex, the dwell time might be affected, leading to a change in *p*_off_ and the number of steps per association.

When we tried to extract microscopic parameters such as the probability of enzyme loss from an internal or a terminal position of linear DNA substrates, we found that a simple random one-dimensional walk model underestimates the vvUNG transfer at short (~20 nt) intersite distances. While the value of *p*_off_ produced by the numerical simulation is reasonable, being severalfold higher than that obtained for EcoUng, the higher-than-expected *P*_T_ at shorter distances may indicate the existence of additional processes beyond one-dimensional sliding. A similar phenomenon was observed by Porecha and Stivers for EcoUng [37] and was taken as evidence that hopping dominates over sliding at distances of >5–10 nt. Moreover, the lack of strand dependence of cleavage [37] and inhibition of correlated cleavage by free uracil [43] are also more compatible with the hopping rather than sliding mechanism, assuming that enzyme reorientation on DNA or inhibitor binding cannot occur in the sliding mode. We also added short-distance hopping (with exponentially or power-law scaled probability) or pausing (essentially increasing the dwell time) to the walk scheme but this did not improve the outcome, with the hopping or pausing probabilities converging to nearly zero, while greatly lengthening the simulation. Hence, we did not pursue more complicated models at the time, and the relative contributions of sliding and hopping mechanism in the correlated target search by vvUNG remain to be addressed. Indirectly, the ability of vvUNG to traverse short gaps and the residual correlated cleavage in the presence of a bulky adduct (Figure 2) suggest that hopping indeed contributes to the lesion search. Interestingly, judging from the *p*_end_ values, vvUNG seems to be much less prone to a loss from the end of the duplex than EcoUng. Although this may be an artifact of the random walk model, the high affinity of vvUNG for DNA ends could be biologically meaningful, because the viral replicative complex has to remain or reassemble at the linear DNA terminus during reinitiation of self-priming hairpin replication [80,81].

One question relevant to vvUNG processivity is whether the isolated vvUNG protein is in the same form as vvUNG in the replicative complex. There is a certain discrepancy in the literature regarding the stoichiometry of vvUNG. The early structural studies of the isolated protein suggested that vvUNG exists as a dimer, and two dimerization interfaces were seen in crystal structures [22,23,25]. Notably, two interacting vvUNG molecules were observed in the crystal structure of a complex with undamaged DNA [28]. However, the most extensive interface observed in the isolated vvUNG homodimer was occupied by A20 in the replicative complex [26,29], and the 1:1:1 stoichiometry of binding of vvUNG, A20, and DNA polymerase [82,83] strongly suggests that the functional form of vvUNG as a processivity subunit is a monomer. In a recently determined cryo-EM structure of the monkeypox virus replicative complex, the three subunits were also found in a 1:1:1 ratio [84]. All other known DNA glycosylases are monomeric, and direct measurement of vvUNG mass in solution by analytical ultracentrifugation and size-exclusion chromatography suggests that the dimer appears only at high protein concentrations (~10 mg/mL) [26,30]. Thus, the functional glycosylase form of vvUNG in the cell and in our experiments is also likely monomeric.

Several amino acid substitutions in vvUNG are known to reduce the protein’s ability to support processive DNA synthesis while retaining DNA glycosylase activity [23]. All of them affect basic residues located at the DNA-binding surface of the protein but distant from the active site pocket and are thus expected to perturb non-specific protein–DNA electrostatic interactions. The G179R mutation at the vvUNG/A20 interface also destabilizes the interaction and results in a loss of processive DNA synthesis [34]. It appears that both the processivity of the vvUNG subunit and its proper coupling with the polymerase through A20 are important for the replication complex processivity.

The interface between vvUNG and the bridging A20 component of the poxviral replisome is an attractive target for the development of protein–protein interaction disruptors, a class of drugs actively pursued at present [85]. A number of hits that interfere with UNG from several poxviral species binding to A20 have been identified [86,87,88,89,90]. Notably, the assay used for the discovery of these molecules is based on the ability of the viral DNA polymerase to incorporate labeled dNMPs, which confirms the importance of the vvUNG/A20 interaction for the functionality of the polymerase complex. Our results, which show there is a possibility of direct suppression of the vvUNG processivity by low-molecular-weight compounds, point to an alternative mechanism that could be exploited to impede poxviral replication.

It remains to be seen how our in vitro quantitative results translate into the processivity of vvUNG and the viral replicative complex in vivo. Studying the mechanisms of lesion search in living cells is extremely challenging due to the fast timescale of the process, and only a handful of reports have been published for any DNA glycosylase in either bacteria or eukaryotes [51,91]. It is not known whether all vvUNG is sequestered in the viral replisome or exists partially as a free repair protein. Given that the vaccinia virus genome is AT-rich and, in addition to vvUNG, encodes a dUTPase [80,81], uracil may be an intrinsic problem for the virus, which vvUNG, with its efficient damage search mechanism, helps to alleviate in either a replication-coupled or replication-uncoupled manner. Our results add to the growing body of data suggesting that vvUNG may act as a viral replication processivity factor. In comparison with ubiquitous processivity clamps, vvUNG may seem less efficient: the size of the vaccinia virus genome is ~195 kb, so even the ~21 kb per association processivity estimate (see above) is likely insufficient for the replication complex to fully copy the genome without subunit exchange. On the other hand, poxviruses replicate in cytoplasmic factories, where the restricted volume might increase the concentration of vvUNG and the polymerase and facilitate reloading of the replicative complex to the released primer end, or re-association of isolated vvUNG to the scanned DNA. To our knowledge, there has been no direct estimate of vaccinia virus or other poxvirus replication processivity in vivo. Recently developed fluorescence-based methods allowing observation of polymerase exchange in living cells [92] could benefit the studies of viral replication processivity and clarify the role of vvUNG.

## 4. Materials and Methods

### 4.1. Enzymes, Oligonucleotides, and Chemicals

Restriction endonucleases and T4 DNA ligase were from Thermo Fisher Scientific (Waltham, MA, USA). Oligonucleotides were synthesized in-house from commercially available phosphoramidites (Glen Research, Sterling, VA, USA). The sequences are listed in Table 2. Oligonucleotides were 5′-labeled using γ[^32^P]-ATP (ICBFM Laboratory of Biotechnology, Novosibirsk, Russia) and T4 polynucleotide kinase (Biosan, Novosibirsk, Russia). Inhibitors were purchased from Vitas-M Chemical Ltd. (Hong Kong, China).

### 4.2. vvUNG Cloning and Purification

The *D4R* gene was cloned from the LIVP vaccinia virus strain (a derivative of the Lister strain) from the collection of the State Research Center of Virology and Biotechnology Vector [93]. The virus was grown on the CV-1 culture of African green monkey kidney cells, DNA was isolated using the QIAamp DNA Mini Kit (Qiagen, Venlo, The Netherlands), and the target gene was amplified using the pair of primers D4Rfwd and D4Rrev (Table 2). The PCR product was subcloned into pBluescript II SK(−), verified by sequencing, and the insert was recloned into the pET-15b expression vector at NdeI and XhoI sites. The resulting His_6_-tagged vvUNG (see Supplementary Text) was overexpressed in BL21(DE3) *E. coli* strain. The culture was grown in LB broth containing 100 μg/mL ampicillin at 37 °C until A_595_ = 0.8, then shifted to 25 °C and induced overnight with 0.5 mM isopropyl β-D-1-thiogalactopyranoside. All subsequent steps were conducted at 4 °C. The cells were harvested by centrifugation, the pellet was resuspended in the lysis buffer consisting of 10 mM Tris–HCl (pH 8.0), 1 mM EDTA, 500 mM NaCl, and 1 mM phenylmethylsulfonyl fluoride, and disrupted by sonication. The lysate was clarified by centrifugation at 15,000× *g* for 20 min. The supernatant was treated with ammonium sulfate at 60% saturation for 2 h and precipitated at 15,000× *g* for 20 min. The pellet was dissolved in Buffer A consisting of 20 mM Tris–HCl (pH 7.5) and 500 mM NaCl. The solution was filtered using 0.45 μm syringe filters (MilliporeSigma, Burlington, MA, USA), loaded onto a 5 mL HisTrap column (GE Healthcare, Chicago, IL, USA) equilibrated in the same buffer, and purified using a 50–500 mM imidazole gradient. The fractions containing the target protein were identified by 12% SDS-PAGE (Laemmli system). The purest fractions, estimated as >95% homogeneous by Commassie Blue staining (Supplementary Figure S6), were pooled, aliquoted and stored at −80 °C until use.

### 4.3. Substrate Preparation

For the steady-state kinetics on U20L and U20 halves, these were ^32^P-labeled and annealed to a 1.5-fold molar excess of their respective complementary strands (G20L and G20R). Internally ^32^P-labeled substrates for the correlated cleavage assay were prepared as described previously [35,41] using the same general scheme: the right half of the substrate (U20R) was ^32^P-labeled at the 5′-end, mixed with a 1.5-fold molar excess of the left half (U20 or U21), the linker strand as needed (L21, L41, or L61), and one or two complementary strands (G40, G41, G61, G41L, G40R, G50L, G51R), depending of the length of the final substrate. After ligation, the duplexes were purified by non-denaturing electrophoresis in 8% or 12% polyacrylamide gel, desalted on a C18 NenSorb column (DuPont, Wilmington, DE, USA) and re-annealed. To prepare single-stranded U20L–U20R for gapped DNA experiments, the longer G46 strand was used as a ligation scaffold to ensure its complete separation by electrophoresis in a denaturing 20% polyacrylamide gel/8 M urea. Structures of all substrates are shown in Supplementary Figure S7.

### 4.4. Steady-State Kinetics

The reaction mixture contained 50–2500 nM ^32^P-labeled substrate (U20L//G20L or U20R//G20R), 25 mM Tris–HCl (pH 7.5), 1 mM EDTA, 1 mM DTT, 0.1 mg/mL bovine serum albumin (BSA), and 0.12 nM vvUNG. After 5 min at 37 °C, aliquots were withdrawn, quenched by adding NaOH to 200 mM, heated for 2 min at 95 °C, neutralized with an equimolar amount of HCl, mixed with an equal volume of the gel loading solution (90% formamide, 0.05% bromophenol blue, 0.05% xylene cyanol), and heated again for 5 min. The reaction products were resolved by electrophoresis in 20% polyacrylamide gel/8 M urea and visualized by posphorimaging (Typhoon FLA 9500 scanner, GE Healthcare). Steady-state kinetic parameters (*K*_M_ and *k*_cat_) were calculated from three independent experiments by non-linear fitting to a Michaelis–Menten equation using SigmaPlot v11.0 (Systat Software, Chicago, IL, USA).

### 4.5. Correlated Cleavage Assay

The correlated cleavage assay protocols were similar to those described in [35,41]. The reaction mixture contained 50 nM substrate, 25 mM Tris–HCl (pH 7.5), 1 mM EDTA, 1 mM DTT, and 0.1 mg/mL BSA. Additionally, the mixture contained 10–200 mM KCl or 5–20 mM MgCl_2_ in the salt dependence experiments, and an inhibitor (10, 100 or 1000 μM) in the inhibition experiments. The reaction was initiated by adding vvUNG to 0.12 nM. After 0.5, 1, 1.5, 2, 3, 5, 7 and 10 min at 37 °C, aliquots were withdrawn and processed as described above. Initial reaction velocities were determined from the initial slopes of the reaction curves. The probability of correlated cleavage (see Supplementary Text) was estimated from three independent experiments as *P*_cc_ = *v*_P3_/(*v*_P1_ + *v*_P2_ + *v*_P3_), where *v*_P1_ and *v*_P2_ are rates of accumulation of the products of cleavage at one of the U sites, and *v*_P3_ is the rate of accumulation of the product of cleavage at both sites [35].

### 4.6. Quench-Flow Experiments

The reactions were performed in a three-syringe rapid chemical quench apparatus RQF3 (KinTek, Snow Shoe, PA, USA) at 37 °C. The reaction mixture contained 20 nM ^32^P-labeled U20R//G20R substrate, 20 mM Tris–HCl (pH 7.5), 1 mM EDTA, 1 mM DTT, 0.1 mg/mL BSA, and 2 μM vvUNG. The equal volumes of the enzyme and the substrate pre-diluted in the same buffer were mixed, and after 2.5 ms the reaction was quenched with 200 mM NaOH or chased with 3.8 mg/mL heparin. The reactions with the chaser were quenched in 5, 10, 15, or 20 s with 200 mM NaOH, then all the tubes were heated for 5 min at 95 °C and neutralized with an equimolar amount of HCl. The fractions were evaporated down to ~20 μL, and the reaction products were resolved, visualized, and quantified as above. All time points were repeated 3–5 times.

### 4.7. Microscale Thermophoresis

All reaction mixtures with a final volume of 10 μL consisted of 50 nM Cy3-labeled oligonucleotide ligand, 0.17–30 μM vvUNG, 20 mM Tris–HCl (pH 7,5), 50 mM KCl, 1 mM EDTA, 1 mM DTT, 5% (*v*/*v*) glycerol, and 0.05% Tween 20. Measurements were carried out using standard capillaries in the Monolith NT.115 device (NanoTemper Technologies, Munich, Germany) in a red/green detection channel at medium infrared laser power. The data were fitted to a one-site ligand binding model using SigmaPlot v11.0.

### 4.8. One-Dimensional Walk Simulation

On a finite one-dimensional grid of length *L* (*L* = 40, 41, 61, 81 or 101) with two selected nodes, A (position 8) and B (position 28, 29, 49, 69 or 89), we modeled a random walk of a single-node-size particle that starts at either A or B, can be lost at each step from an internal node with a probability *p*_off_ and from a terminal node with a larger probability *p*_off_ + *p*_end_ (if *p*_off_ + *p*_end_ > 1, the combined probability of falling off the end in the simulation was taken for 1), and otherwise moves to any of the two adjacent grid nodes at each step with an equal chance. At the first iteration, a 6 × 6 matrix with *p*_off_ and *p*_end_ values evenly spaced between 0 and 1 was generated. For each *p*_off_, *p*_end_ pair we simulated 10,000 walks from A to B and 10,000 walks from B to A until either success (a completed walk between the selected nodes) or loss, and counted the number of successes for each bin of 100 walks. Thus, for every *p*_off_, *p*_end_ pair 200 simulated transfer probabilities *P*_Tsim_ were obtained. The *p*_off_, *p*_end_ pair producing the least sum of squares of differences from the experimentally determined *P*_T_ values, *S* = Σ(*P*_Tsim_ − *P*_T_)^2^, over all simulations for all five grids, was carried into the next round, and the *p*_off_ and *p*_end_ values adjacent to the best pair in the matrix were taken as the bounds to generate a new 6 × 6 matrix. The iterations were continued until the standard deviation of all *S* values in the matrix was less than 0.1 of *S* for the best *p*_off_, *p*_end_ pair, which took 7–8 rounds. Ten such simulations were carried out, and the mean of the produced *p*_off_ and *p*_end_ values were taken as the final estimates. Lastly, with these *p*_off_ and *p*_end_, 10,000 walks from A to B and 10,000 walks from B to A were simulated as above to obtain a distribution of *P*_Tsim_. The whole procedure was implemented as a Python script available at github.com/mizarium/MC_walk.

## Figures and Tables

**Table 1:** vvUNG affinity for normal and damaged DNA.

Substrate (Oligo IDs)	*K*_d_, µM ^a^
C (13C)	7.0 ± 2.0
T (13T)	12 ± 7
F (13F)	4.0 ± 2.0
C:G (13C//13cmpG)	7.2 ± 1.5
T:A (13T//13cmpA)	8.7 ± 1.7
F:G (13F//13cmpG)	6.2 ± 2.3
F:A (13F//13cmpA)	6.7 ± 1.4

^a^ Mean ± SD of three independent experiments.

**Table 2:** Oligonucleotides used in this study.

Oligo ID	Sequence (5′ → 3′)
*D4R* gene cloning	
D4Rfwd	GGCATATGAATTCAGTGACTGTATC
D4Rrev	GGGGATCCTAAAATTTCACTAAGC
Processivity studies	
U20L	TCCCTTCUCTCCTTTCCTTC
U20R	GGACTTCUCTCCTTTCCAGA
U21L	TCCCTTCUCTCCTTTCCTTCC
U40F ^1^	TCCCTTCUCTCCTTTCCTTC[FluoT]GACTTCUCTCCTTTCCAGA
G17L	GGAAAGGAGGGAAGGGA
G17R	TCTGGAAAGGAGGGAAG
G18L	AGGAAAGGAGGGAAGGGA
G18R	TCTGGAAAGGAGGGAAGT
G19L	AAGGAAAGGAGGGAAGGGA
G19R	TCTGGAAAGGAGGGAAGTC
G20L	GAAGGAAAGGAGGGAAGGGA
G20R	TCTGGAAAGGAGGGAAGTCC
G40	TCTGGAAAGGAGGGAAGTCCGAAGGAAAGGAGGGAAGGGA
G40R	TCTGGAAAGGAGGGAAGTCCGAGGTCTGAACGAGAGGAAA
G41	TCTGGAAAGGAGGGAAGTCCGGAAGGAAAGGAGGGAAGGGA
G41L ^2^	[p]GATCGCACAAATGAAAGGTCCGAAGGAAAGGAGGGAAGGGA
G46	TTTTCTGGAAAGGAGCGAAGTCCGAAGGAAAGGAGCGAAGGGATTT
G50L	[p]AAATTCACTCATCGCACAAATGAAAGGTCCGAAGGAAAGGAGGGAAGGGA
G51R	TCTGGAAAGGAGGGAAGTCCGAGGTCTGAACGAGAGGAAAGCTAAATCCCG
G61	TCTGGAAAGGAGGGAAGTCCGCTCTAACGCAAGTAAAGTCCGAAGGAAAGGAGGGAAGGGA
L21	[p]GGACTTTACTTGCGTTAGAGC
L41	[p]GGACCTTTCATTTGTGCGATCTTTCCTCTCGTTCAGACCTC
L61	[p]GGACCTTTCATTTGTGCGATGAGTGAATTTCGGGATTTAGCTTTCCTCTCGTTCAGACCTC
Microscale thermophoresis	
13C ^3^	[Cy3]CCTTCCCTCCTTT
13T	[Cy3]CCTTCTCTCCTTT
13F	[Cy3]CCTTCFCTCCTTT
13cmpG	AAAGGAGGGAAGG
13cmpA	AAAGGAGAGAAGG

^1^ [FluoT], fluorescein-dT ^2^ [p], synthetically introduced 5′-phosphate ^3^ [Cy3], synthetically introduced 5′-Cy3 dye.

## Data Availability

The script for modelling one-dimensional walks can be accessed at github.com/mizarium/MC_walk (accessed on 20 May 2023). All other data are provided in the paper and the Supplementary Materials.

## Supplementary Materials

The following supporting information can be downloaded at https://www.mdpi.com/article/10.3390/ijms24119113/s1. References [94,95] are part of the Supplementary Materials.
