# Supplementary material for: Correlated Target Search by Vaccinia Virus Uracil–DNA Glycosylase, a DNA Repair Enzyme and a Processivity Factor of Viral Replication Machinery
Source: Int J Mol Sci. 2023 May 23;24(11):9113. doi: 10.3390/ijms24119113 (PMC10253040; doi:10.3390/ijms24119113)
Supplement: Supplementary file 1 [file ijms-24-09113-s001.zip › ijms-2383574-supplementary.pdf]

International Journal of *Molecular Sciences*

MDPI

Supplementary material for

Correlated target search by vaccinia virus uracil–DNA glycosylase, a DNA repair enzyme and a processivity factor of viral replication machinery

Evgeniia A. Diatlova [1], Grigory V. Mechetin [1], Anna V. Yudkina [1], Vasily D. Zharkov [2], Natalia A. Torgasheva [1], Anton V. Endutkin [1], Olga V. Shulenina [3], Andrey L. Konevega [3], Irina P. Gileva [4], Sergei N. Shchelkunov [4] and Dmitry O. Zharkov [1,5,*]

1 SB RAS Institute of Chemical Biology and Fundamental Medicine, 8 Lavrentieva Ave., 630090 Novosibirsk, Russia; e.diatlova@g.nsu.ru (E.A.D.); mechetin@niboch.nsc.ru (G.V.M.); ayudkina@niboch.nsc.ru (A.V.Y.); ashatan.314@gmail.com (N.A.T.); aend@niboch.nsc.ru (A.V.E.); dzharkov@niboch.nsc.ru (D.O.Z.)
2 Tomsk State University, Tomsk, 634050, Russia; arthropodae01@gmail.com (V.D.Z)
3 NRC "Kurchatov Institute" – B. P. Konstantinov Petersburg Nuclear Physics Institute, Gatchina, Leningrad Region, 188300, Russia; ovshulenina@gmail.com (O.V.S.); konevega_al@pnpi.nrcki.ru (A.L.K)
4 State Research Center of Virology and Biotechnology Vector, Koltsovo, Novosibirsk Region, 630559, Russia; gileva@vector.nsc.ru (I.P.G.); snshchel@rambler.ru (S.N.S)
5 Department of Natural Sciences, Novosibirsk State University, 2 Pirogova St., 630090 Novosibirsk, Russia
* Correspondence: dzharkov@niboch.nsc.ru; Tel: +7 383 363 5187; Fax: +7 383 363 5128

This file contains

- Supplementary Text
- Supplementary Figures S1–S7

1. Supplementary Text

1.1. Properties of the recombinant vvUNG

Protein sequence of the cloned vvUNG and calculated protein parameters [89]. The sequence from the pET-15b vector is shown in bold and underlined.

>vvUNG
<u>MGSSHHHHHHSSGLVPRGSH</u>MNSVTVSHAPYTITYHDDWEPVMSQLVEFYNEVASWLLRD
ETSPIPDKFFIQLKQPLRNKRVCVCGIDPYPKDGTGVPFESPNFTKKSIKEIASSISRLT
GVIDYKGYNLNIIDGVIPWNYYLSCKLGETKSHAIYWDKISKLLLQHITKHVSVLYCLGK
TDFSNIRAKLESPVTTIVGYHPAARDRQFEKDRSFEIINVLLELDNKAPINWAQGFIY

Number of amino acids	238
Molecular weight	27215
Theoretical pI	8.25
Extinction coefficient	45380 $M^{-1}\times cm^{-1}$, assuming all Cys are reduced

1.2. Probability of correlated cleavage formalism

The probability of correlated cleavage of a two-site substrate (Fig. 1A in the main text) is defined as the conditional probability of cleavage at the second site (P3 formation) given the first site is already cleaved (P1, P2 formed). Under the conditions of substrate excess and assuming the probability of transfer between them is the same in either direction, cleavage of the substrate containing two equivalent target sites can be described by the following kinetic scheme:

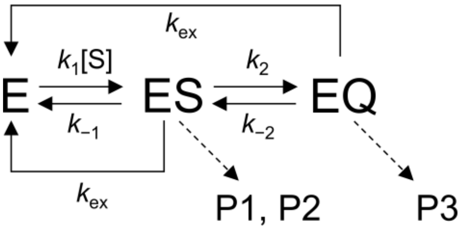

where k_1 is the second-order rate constant of enzyme binding to either target site, k_{-1} is the substrate release rate constant (without cleavage), k_{ex} is the cleavage rate constant, k_2 is the rate constant of transfer between two sites after the cleavage at the first site but without product release, and k_{-2} is the rate constant of transfer between two sites without the cleavage (formally, the process described by k_2 and k_{-2} may be regarded as isomerization of the ES complex). Applying the graph theory formalism [90], the determinants for different enzyme complexes can be written as:

$$\Delta_{E} = k_{-1}k_{-2} + k_{ex}^{2} + k_{-1}k_{ex} + k_{-2}k_{ex}$$
$$\Delta_{ES} = k_{-2}k_{1}[S] + k_{ex}k_{1}[S]$$
$$\Delta_{EQ} = k_{1}k_{2}[S]$$

Hence, the ratio of P3 product formation to the combined rate of P1, P2 and P3 formation will be:

$$\frac{v_{P3}}{(v_{P1} + v_{P2}) + v_{P3}} = \frac{\Delta_{EQ}}{\Delta_{ES} + \Delta_{EQ}} = \frac{k_1 k_2[S]}{k_{-2}k_1[S] + k_{ex}k_1[S] + k_1 k_2[S]} = \frac{k_2}{k_{-2} + k_{ex} + k_2}$$

Assuming $k_{-2} \ll k_{ex}$ (i. e., the probability of cleavage upon reaching the second target site greatly exceeds the probability of return back to the first site), one finally has:

$$\frac{v_{P3}}{(v_{P1} + v_{P2}) + v_{P3}} = \frac{k_2}{k_{ex} + k_2} \equiv P_{cc}$$

It should also be noted that P_{cc} is not a function of the enzyme concentration. Thus, the presence of competitive inhibitors does not affect P_{cc}, since they decrease the concentration of the free enzyme and thus decelerate all reaction rates to the same extent.

2. Supplementary Figures

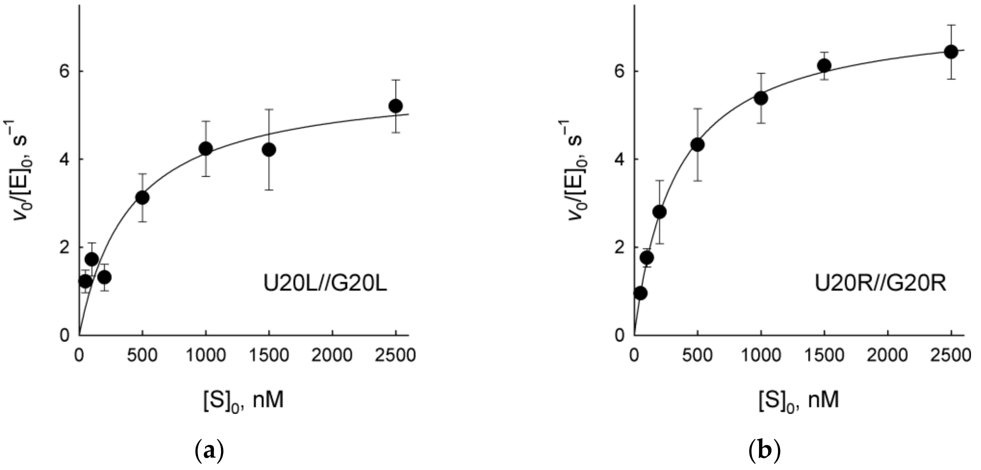

Supplementary Figure S1. Michaelis–Menten kinetic plots of cleavage of 20-mer duplex substrates corresponding to each individual half of the ligated 40-mer construct by vvUNG. (**a**) U20L//G20L substrate; (**b**) U20R//G20R substrate. Mean ± SD of three independent experiments is shown.

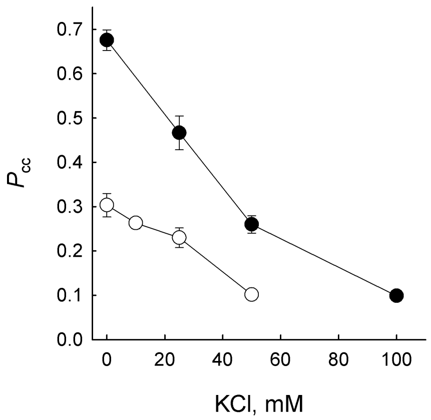

Supplementary Figure S2. Comparison of the dependence of vvUNG and EcoUng P_{cc} on the concentration of KCl. Closed circles, vvUNG; open circles, EcoUng (data from [41]). Mean ± SD of three independent experiments is shown.

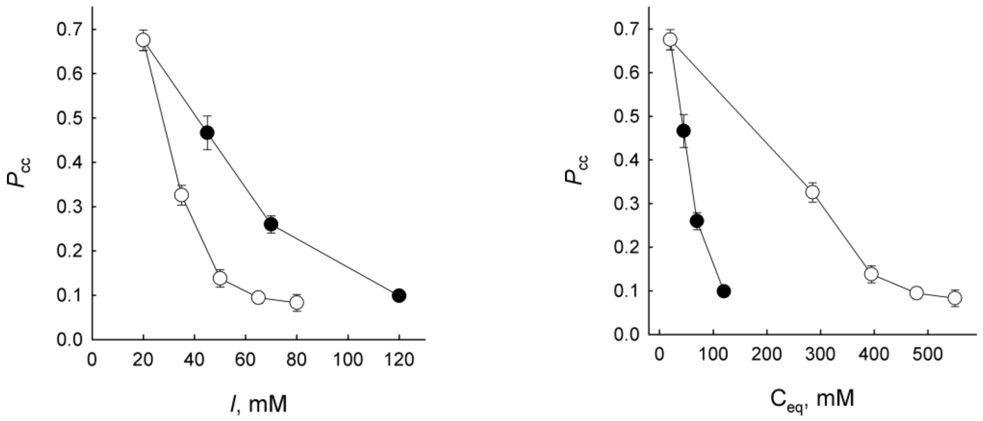

(**a**) (**b**)

Supplementary Figure S3. Dependence of vvUNG P_{cc} on the ionic strength and the equivalent monovalent cations concentrations of Tris–HCl buffer supplemented with KCl or $MgCl_2$. (**a**) Plot of P_{cc} vs the ionic strength. (**b**) Plot of P_{cc} vs the equivalent monovalent cations concentrations. Closed circles, KCl; open circles, $MgCl_2$. C_{eq} was calculated from an empirical equation $C_{eq} = [Mono^+] + 3.75 \times \sqrt{[Mg^{2+}]}$, where $[Mono^+]$ is total concentration of monovalent cations in the buffer and all concentrations are in mol/L [57]. Mean ± SD of three independent experiments is shown.

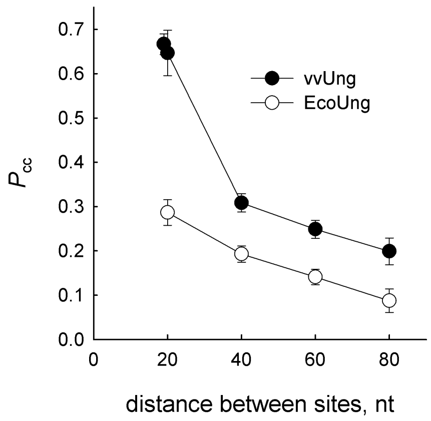

Supplementary Figure S4. Comparison of the dependence of vvUNG and EcoUng P_{cc} on the distance between the Ura residues. Closed circles, vvUNG; open circles, EcoUng (data from [41]). Mean ± SD of three independent experiments is shown.

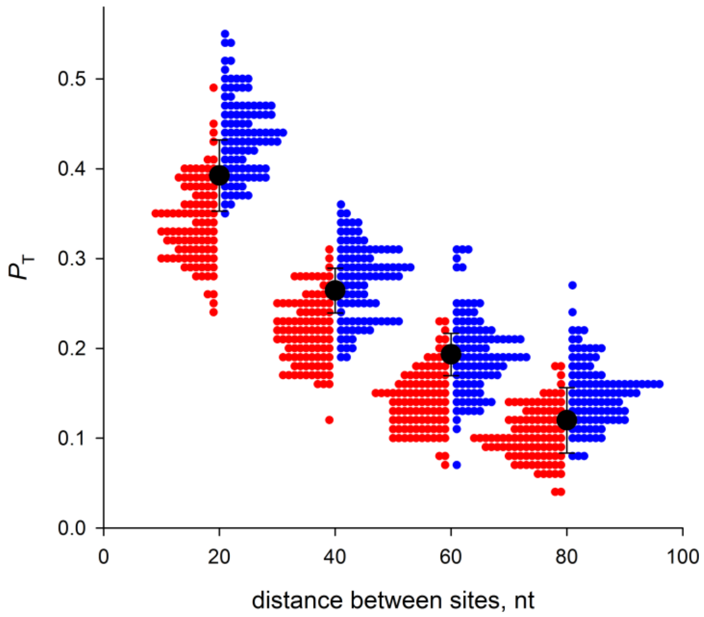

Supplementary Figure S5. Simulation of EcoUng random walk on a finite one-dimensional grid with irreversible losses. Black symbols are experimental P_T data from [41] used for fitting. Colored dots show the fraction of successful walks from position 8 to positions 29, 49, 69 or 89 (red dots) or from positions 29, 49, 69 or 89 to position 8 (blue dots).

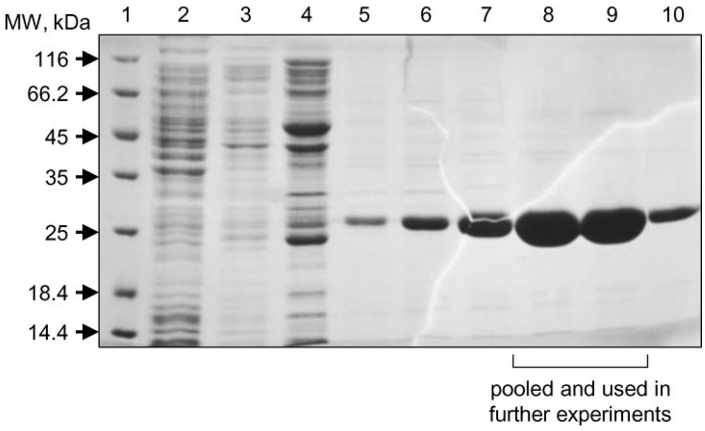

Supplementary Figure S6. Purification of vvUNG. Lane 1, molecular weight markers; lane 2, HisTrap column flowthrough; lane 3, wash with Buffer A; lane 4, wash with Buffer A supplemented with 50 mM imidazole; lanes 5–10, fractions under the major UV absorption peak. Fractions from lanes 8 and 9 were pooled and used in further experiments.

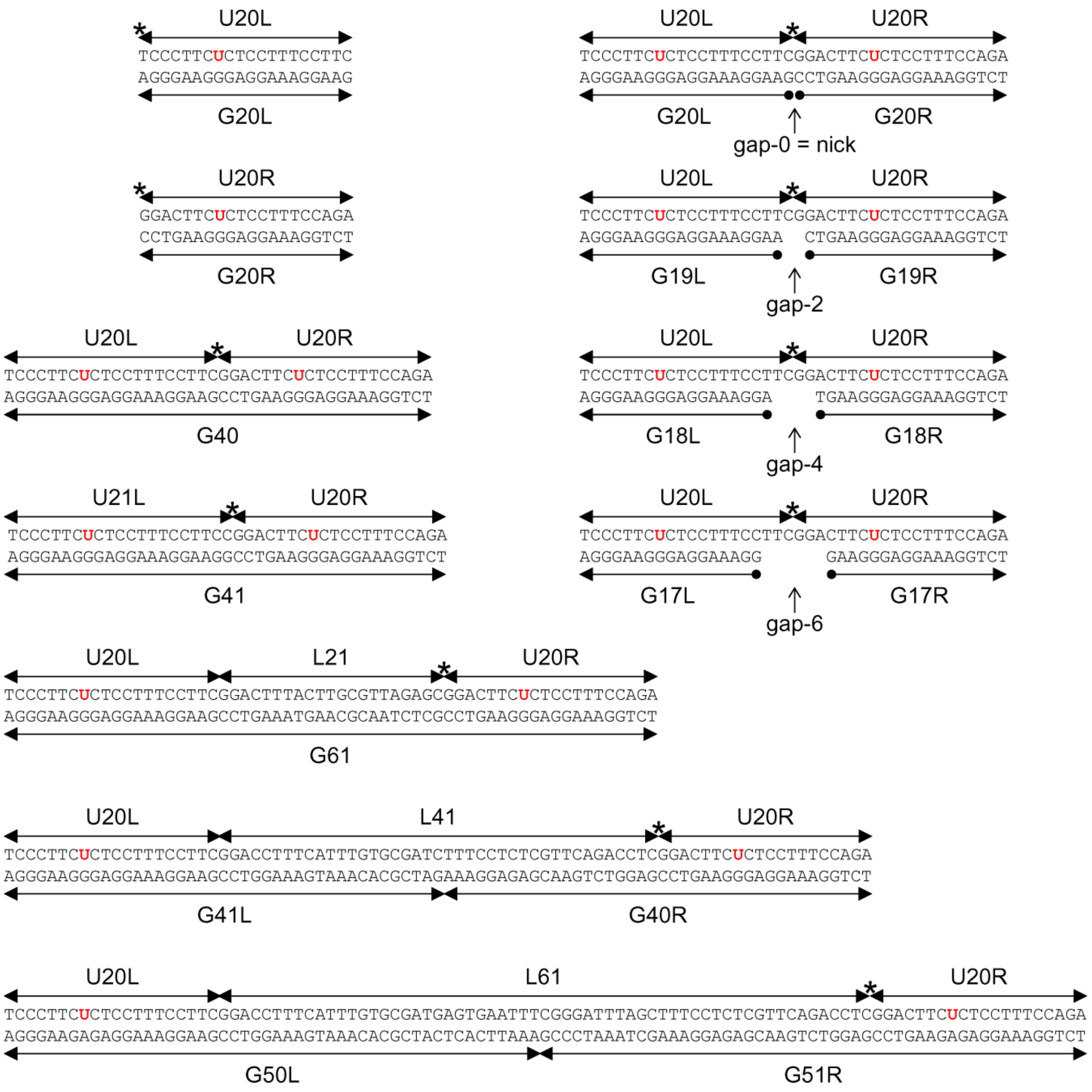

Supplementary Figure S7. Scheme of substrates construction. The target uracil residues are shown in red. Asterisk indicates the placement of the radioactive label.
